# Patient acceptance and outcome of mental health screening in Swedish adults with cystic fibrosis

**DOI:** 10.1007/s11136-020-02417-5

**Published:** 2020-01-09

**Authors:** Stina Järvholm, Petrea Ericson, Marita Gilljam

**Affiliations:** 1 Department of Obstetrics and Gynecology, Institute of Clinical Sciences, Sahlgrenska University Hospital, Sahlgrenska Academy, University of Gothenburg, Gröna stråket 9, 413 45 Gothenburg, Sweden; 2 Department of Internal Medicine and Clinical Nutrition, Institute of Medicine, Sahlgrenska University Hospital, Respiratory Medicine, Sahlgrenska Academy, University of Gothenburg, Gothenburg, Sweden

**Keywords:** Anxiety, Cystic fibrosis, Depression, Patient acceptance of health care

## Abstract

**Purpose:**

Anxiety and depression are common among adults with cystic fibrosis (CF), and the International Committee on Mental Health in CF (ICMH) recommends annual screening for mental health problems. We implemented screening according to the recently published guidelines and assessed the results from the first year, as well as the patients’ attitude to annual screening

**Methods:**

Adult patients attending Gothenburg CF-center from Feb 2015 to Dec 2016 completed the GAD-7 (anxiety) and PHQ-9 (depression) forms at the time of their annual review. In addition, questions regarding the screening process and instruments used were asked.

**Results:**

All invited patients (*n* = 100, 52% males, 2% lung transplanted), with a median age of 28 years (range 18–65), agreed to participate. In general (83%), the patients were positive to screening on an annual basis. No significant differences in total GAD-7 and PHQ-9 scores were found when comparing men and women. Patients younger than 30 years of age reported more symptoms of anxiety compared to older patients (*p* = 0.02). There were 21 (21%) patients with scores > 10 for GAD-7 and/or PHQ-9 indicating at least moderate anxiety or depression. Scores > 10 were reported by 15 patients on GAD-7, 15 patients on PHQ-9, and 9 patients reported scores above 10 on both measures.

**Conclusion:**

The patients considered annual check-ups for mental health issues important. Although the screening results are reassuring, the group is heterogenic and younger individuals should be given extra attention. Follow-up over longer time will provide more robust data.

## Introduction

Cystic fibrosis (CF) is a progressive, genetic disease with symptoms in many organs and a shortened expected life span [1]. Mutations in the cystic fibrosis transmembrane conductance regulator (CFTR) gene cause a dysfunctional CFTR protein, which functions as a chloride channel in cell membranes. As a consequence, the mucus in various organs becomes thick and sticky. Severe, persistent, bacterial lung infection leading to respiratory failure is the major cause of death in this patient population. Liver cirrhosis, diabetes mellitus, sinus disease, and infertility are other CF-related complications. The necessary, daily treatment is time-consuming and cumbersome and includes inhalation of bronchodilators and mucus-solving drugs, mucus-mobilization, and physical exercise. The chronic airway infection is treated with oral, inhaled, and intravenous antibiotics. Survival has increased dramatically and the majority of people with CF are now adults [2]. Centralized care with multidisciplinary care teams, improved drugs, and focus on prevention and early treatment of complications are key success factors. Lung and liver transplantations are established treatment options in severe disease and the development of CFTR modulating drugs gives hope for the future [3, 4]. CF is however still a severe disease requiring a demanding treatment, and access to specialized health care and new drugs is a challenge in many countries [5].

Anxiety and depression are common symptoms among adults with CF [6–8]. A multicenter study including nine countries (154 centers/4739 adults), using the Hospital Anxiety and Depression Scale (HADS) and the Center for Epidemiologic Depression Scale (CES-D), revealed elevated symptoms of depression (19%) and anxiety (22%), with a comorbidity of 14%, among adults with CF [6]. Depression is the most frequent psychiatric diagnosis for individuals experiencing psychological distress in high-income countries [9]. Depression and anxiety cover a broad spectrum of suffering, from individuals being mildly affected in their everyday life to severe conditions. To react with anxiety or depression is expected and common when faced with severe life events. Anxiety and depression are fairly common in the general population and comorbidity is high 28.2% [10]. In a recent study, using the Patient Health Questionnaire 9-item scale (PHQ-9) and the Generalized Anxiety Disorder 7-item scale (GAD-7), Johansson et al. [10] found that the point prevalence of depression and generalized Anxiety Disorder in Sweden was 5.2% and 8.8%, respectively. Women, both CF and non-CF, tend to report higher levels than men regarding both depression [7] and anxiety [8]. The understanding of mental illness is complex and two previous Swedish studies did not indicate an elevated risk for anxiety and depression among adults with CF compared with the general Swedish population [11, 12]. These studies used HADS and the General Health Questionnaire (GHQ-28) to measure anxiety and depression. The HADS, GHQ-28, and CES-D instruments are considered less sensitive for depression compared to PHQ-9, which is the recommended tool for screening in the CF-population today [13]. The previous studies may thus have underestimated depression.

The guidelines published in 2016 recommend annual screening for mental health problems in patients with CF [13]. Little is known about the circumstances regarding implementation of the screening. In a recent survey, 50% of the responding CF-centers in Europe (*n* = 187) answered that screening was carried out. The recommended tools were used by 80% of the centers [14]. Using questionnaires is time efficient but may lead to under-reporting of problems. Verkleij et al. [15] found that people with CF reported more and different types of problems during face-to-face conversations with the psychologist than they reported using the screening tools. At the annual European cystic fibrosis congress in Seville 2017, we presented our first experiences and impression of establishing the guidelines [16].

In the present study, our aim was to assess the occurrence of anxiety and depression as well as the patients’ view of annual screening.

## Methods

### Introduction of screening

The new guidelines, recommending annual screening for mental health, were presented to the CF-team. Any obstacles or opposition to screening, as well as the best way to implement the guidelines, were discussed. A strategy was agreed upon and patients were informed in a newsletter of the new guidelines and the planned process. An action plan for patients with unexpected high scores was made. Evaluation of the implementation was scheduled to be performed once the majority of the patients had been screened once.

### Procedure

All adult patients attending the Gothenburg CF-center between February 2015 and December 2016 were consecutively asked to complete the questionnaires regarding anxiety and depression, and three additional questions on patient acceptance, at the time of their annual comprehensive assessment. The majority of the patients were screened within a year while a few patients with a postponed yearly assessment were included during the later study period. The three questions asked about patients’ acceptance were derived from the team’s discussion.

The forms were distributed and collected by the clinic nurses. The psychologist summarized and assessed the scores and wrote a short comment in the medical chart within a week for action when needed. Screening cut-offs of > 10 indicates moderate-to-severe depression. For these patients a personalized plan was created. Additionally, a brief check-in was carried out for patients scoring in the mild range (4–10).

### Measures

Symptoms of depression were measured using the Swedish version of the 9-item Patient Health Questionnaire Depression Scale (PHQ-9) [17]. The PHQ-9 contains 9 items, with a total score ranging from 0 to 27. Total scores of 0–4 indicate no depression, 5–9 mild depression, 10–14 moderate depression, 15–19 moderately severe depression, and 20–27 severe depression [17]. Psychometric properties for the PHQ-9 have been shown to be good, with an internal consistency in the range Cronbach’s α = 0.86–0.89 and a test–retest reliability of *r* = 0.84 [18]. The PHQ-9 is considered a suitable instrument for detecting depression in the general population [19].

Symptoms of anxiety were measured using the Swedish version of Generalized Anxiety Disorder Scale (GAD-7) [20], a 7-item measure with a total score of 21 and a cut-off of 10 to detect anxiety. The GAD-7 has good internal consistency (Cronbach’s α = 0.92) and a good test–retest reliability of *r* = 0.83 [18]. Similar to the PHQ-9, the GAD-7 has been shown to be valid in the general population [21].

The three questions asked regarding the screening program were as follows:

1. Were there questions that you found difficult to answer (yes/no)?
2. Do you think that it is good to answer these questions by questionnaires (yes/no)?
3. How often do you think that these questionnaires should be completed (once a year/when needed/other)?

Comments could be added to each question in free text.

### Statistical methods

Data are presented in numbers, median, and range. The Mann–Whitney *U* test was used for group comparison and the Kendall’s rank correlation coefficient tau, to assess relationships. *p* < 0.05 was used for statistical significance. One young female was excluded from analysis of GAD-7 and PHQ-9 due to multiple unanswered items. Patients’ comments were categorized and structured in domains.

### Ethical aspects

All invited patients agreed to participate. Written informed consent was given prior the study. The Helsinki ethical guidelines [22] were followed and the study was approved by the local ethical committee at the University of Gothenburg Dnr: 1086-17.

## Results

### Patients

From February 2015 to December 2016 all approached patients with CF (*n* = 100, 52 males, one lung- and one liver transplanted), with a median age of 28 years (range 18–65), agreed to participate. The results were evaluated after inclusion of the first 100 patients. At the time, 132 patients attended the clinic and, of the 32 not yet included, 15 were organ transplanted and had their main follow-up at the transplant clinic while 17 individuals either had mild disease and infrequent visits at the CF center or had a postponed assessment. See group comparisons in Table 1.

**Table 1:** Screening results

*n*	Total	Women	Men	< 30 years	≥ 30 years
	100	47*	52	52*	47
Scoring					
GAD-7	3 (0–19)	3 (0–19)	2.5 (0–18)	3 (0–19)**	2 (0–18)
PHQ-9	3 (0–21)	4 (0–20)	3 (0–21)	3.5 (0–21)	3 (0–18)

Data are presented in median (range)

*GAD* Generalized Anxiety Disorder, 7-items, score 0–21. *PHQ* Patient Health Questionnaire, 9-items, score 0–27

*One young female was excluded from analysis due to multiple unanswered items

**Age group comparison, Mann–Whitney test, *p* = 0.02

The median (range) scores were 3 (0–19) for GAD-7 and 3 (0–21) PHQ-9. No significant differences were found in scores for anxiety and/or depression when comparing men and women. Patients younger than 30 years of age reported more symptoms of anxiety compared to older individuals *p* = 0.02.

There were 21 (21%) patients with scores > 10 for GAD-7 (*n* = 15) and/or PHQ-9 (*n* = 15), indicating at least moderate anxiety or depression, or both (*n* = 9). Eight patients scored above 15 for GAD-7 indicating severe anxiety. Eight patients scored > 15 and seven patients scored > 20 for PHQ-9, indicating moderately severe and severe depression, respectively. The majority of patients (14/20) with increased scores were already identified and were receiving psychological interventions. The patients with elevated scores that were not already identified by the team prior to screening were all in the younger age group (< 30 years). There were 19 patients with scores 5–10 for PHQ-9 and 20 patients with scores 5–10 for GAD-7 (no difference for sex or age group), indicating mild reported symptoms of depression and anxiety, respectively. The results were discussed, as needed, with the patients at a follow-up visit at the clinic. There was a moderate high correlation between GAD-7 and PHQ-9; Kendall’s tau = 0.483, *p* = 0.001.

**Fig. 1:**
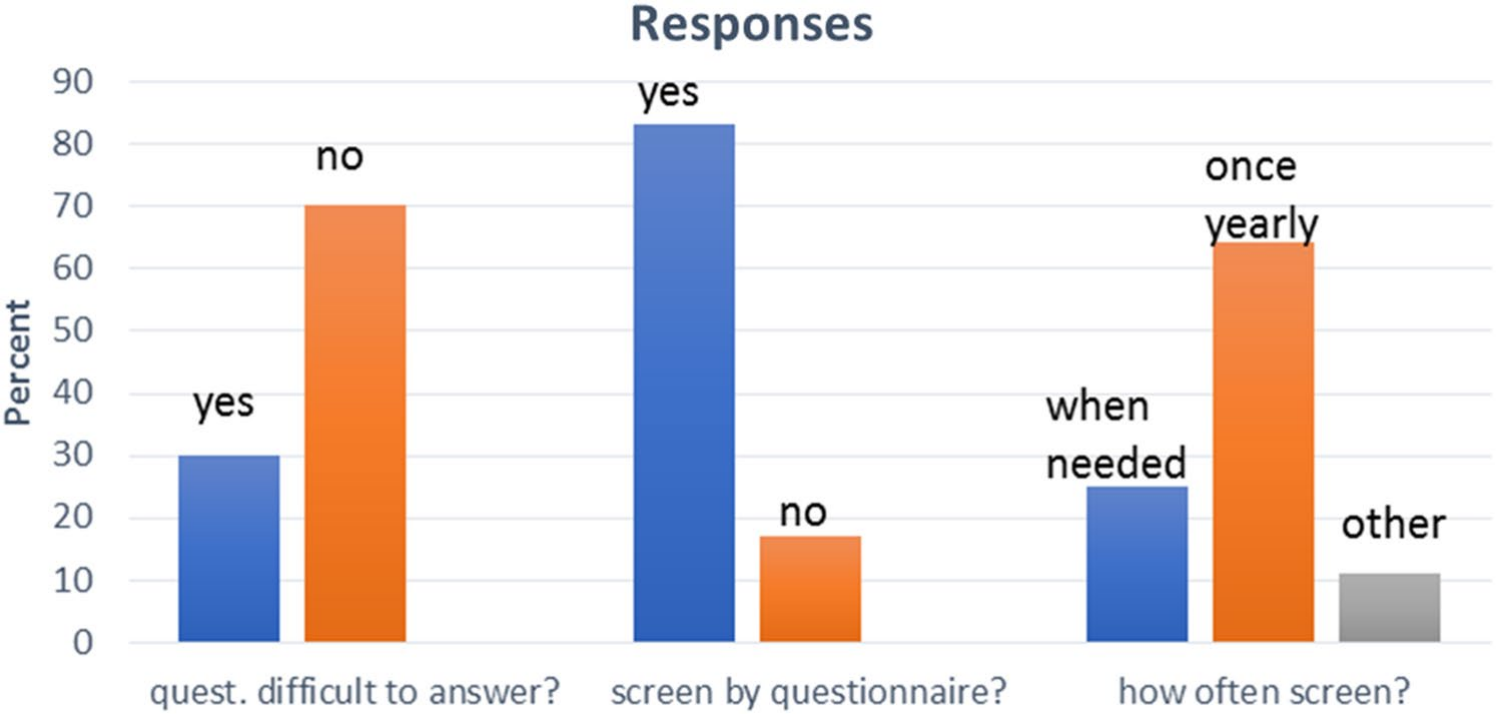
Patients’ evaluation of screening process

In general, the attitude to annual screening was positive (83%) (Fig. 1) and there was no statistically significant difference in responses when comparing age groups and gender (Table 2). Comments in free text were given by 45/100 individuals. Categorization of the patients’ written comments was carried out and structured in three main domains, *positive* (*n* = 13), *constructive thoughts* (*n* = 26), *and negative* (*n* = 6).

**Table 2:** Patients’ evaluation of screening process

	Response	*n*	Yes	No
Where there any questions that were difficult to answer?	All	98	29 (30%)	69 (70%)
	≥ 30 years	47	12 (26%)	35 (75%)
	< 30 years	51	17 (33%)	34 (67%)
Do you think that it is good to answer these questions by questionnaires	All	95	79 (83%)	16 (17%)
	≥ 30 years	46	41 (89%)	5 (11%)
	< 30 years	49	38 (78%)	11 (22%)

			When needed	Once a year	Other
How often do you think you should complete the screening	All	96	24 (25%)	61 (64%)	11 (11%)
	≥ 30 years	45	7 (16%)	32 (71%)	6 (13%)
	< 30 years	51	17 (33%)	29 (57%)	5 (10%)

One hundred adult CF patients (52 men/48 women, 53 < 30 years of age) were asked to complete a three-question evaluation form directly after completing GAD-7 and PHQ-9. Nine patients left 11 questions unanswered. Data are presented as numbers and percent of responding patients

The *positive domain* mainly consisted of general statements such as:

It is good that attention is paid to mental health and that us patients get an opportunity to discuss it

Female 21 years

Of those who had made positive comments 6/13 were women, 6/13 were younger than 30 years and 2/13 had elevated scores (> 10) in at least one questionnaire.

In the *constructive thoughts’ domain* suggestions for improvement of the questionnaires were made.

The difference between the possible answers is too big, for example, an option between “never” and “on several days” would be beneficial

Male 23 years

I would have preferred more detailed questions about what made me feel stressed. For example, is the cause CF, a situation at work or something in my family?.

Male 36 years

Thoughts regarding the screening set-up and interpretation of the answers were also shared.

I think that the timing of the annual screening should vary from year to year since my CF is always much worse at this time of year

Female 27 years

Some expressed that they wanted the screening to be carried out solely verbally at the visit.

I would prefer doing the screening verbally at a visit

Female 42 years

There were 29 individuals who stated that some questions were difficult to answer; 12/29 commented in free text, mainly on the format of the questionnaires. Examples of the comments were “too wide answers,” “too short of a time frame is taken into consideration in the questionnaire,” “answers not related to CF.”

Among those who had written constructive comments, 12/26 were men and 13/26 were younger than 30 years.

The *negative domain* contained comments on the screening method.

These issues are difficult to talk about and should never ever be addressed by a questionnaire

Male 18 years

and about the purpose of screening.

This (screening) doesn’t really add anything, I am already aware of my mental health problems. I would like to add that my experienced strain is not directly connected with CF.

Female 28 years

No group difference was seen for the few patients who wrote negative comments (3 < 30 years, 4 males) and only 2/6 in this group had elevated scores on at least one questionnaire.

## Discussion

Over the last years, the importance of structured mental health screening among people with CF has been highlighted [6–8, 13, 14]. There is limited knowledge of patients’ acceptance of the screening and of possible sub-group differences regarding anxiety and depression. A recent Italian study reported more symptoms of anxiety for adult CF females and mothers to CF children, compared to CF males and fathers [23]. To the best of our knowledge, there are no published studies comparing depression and anxiety between different age groups among the adult population with CF. In our study, 15% of the patients had elevated scores for moderate anxiety and 14% for depression. This is less than the previously reported elevated scores of 19% and 32% for depression and anxiety, respectively [7], but in line with an earlier report of anxiety and depression in a Swedish CF-population [11]. When comparing studies, it is important to consider the differences in study design, questionnaires used, and cut-off values reported. Given the proposed higher sensitivity for the GAD-7 and PHQ-9 compared to for example HADS, our results are reassuring.

We found higher scores for anxiety in the young age group, compared to the older group but overall the results indicated that the majority of the population had no symptoms of anxiety at all. However, anxiety and depression disorders are more common among adolescents and young adults in general [24], in part explained by an increased vulnerability during the biological maturation process as well as all the life changes and decisions for the future that this period of life entails [25, 26]. Living with a chronic disease increases the vulnerability further but, in our opinion, the majority of young people with CF look forward to the anticipated life changes and the transition from pediatric to adult care. Being an adult includes taking responsibility for everyday tasks, in addition to one’s education, economy, well-being, and health. The first severe infection in adult age, or just the sudden insight of how CF restricts daily life and future life perspectives, may cause intense stress and anxiety [27, 28]. The present results are in contrast to a study by Besier, Goldbeck [29] that found life satisfaction to be lower and the burden of the illness to be greater in older patients with CF. We may speculate that easy access to the specialized CF health care team, including psychologist and social worker, and the Swedish social welfare system are protection factors.

Interestingly, and in contrast to the general Swedish population [10], no difference in anxiety or depression was found between men and women with CF. This could have several explanations. In general, a combination of life stress such as adverse life events, chronic stress exposure, poor social support, and limited social networks coupled with limited psychosocial resources is associated with adverse psychological, physical, and quality of life outcomes [30]. In line with this, it is possible that the burden of living with CF could supersede other common reasons for anxiety and depression and, as such, erase the difference between men and women regarding mental health [31]. We found, in contrast to others, no gender difference [6, 23] and will follow-up on this issue in future studies.

Although the used questionnaires have been proven with good validity [18], patients’ comments on the screening tools and whether there are more suitable questionnaires available, should be paid attention to. Overall, the patients were positive to participating in annual mental health screening. Constructive suggestions were in line with the findings by Verkleij et al. [15] that mental health issues might be better discovered during consultation. It might also be a good idea to add a question to the questionnaires asking how the reported symptoms relate to CF. It is reassuring to find that those who gave negative comments did not form a specific group regarding age, gender, or screening results. Those who gave negative feedback were also engaged in the questions and pointed out facts that are important to bear in mind. For example, mental health issues are not always connected to CF and may be better addressed elsewhere. Psychiatric health care or a visit to the general practitioners may sometimes be more appropriate ways to handle mental health problems. The CF psychologist can act as a facilitator by addressing the mental health questions and discussing preferences regarding follow-up.

A previous study found that hesitation in the CF-team to start up mental screening was associated with the fear of lack of referrals or the fear of not being able to manage the possible additional workload connected to the screening or the mental health problems in the patient population [14]. We were concerned about the time needed for implementation, as well as the patients’ thoughts on the planned screening. Our experience is that by involving the whole team in the process and informing the patients ahead of time, we could implement the guidelines in an efficient and reliable way. The extra workload was less than expected. In our opinion, screening by questionnaires saves time and aids the psychosocial team in prioritizing patients. Screening is also possible to be conducted at CF centers with limited access to a psychosocial team.

A limitation of our study is that only two patients having undergone lung transplantation were included. However, for this group, an annual check-up for health and mental health issues is performed at the transplant center. Another limitation is that no health data are included for the group. Symptoms of fatigue or having trouble relaxing could be due both to mental health and/or the CF-disease.

## Conclusion

Annual screening for anxiety and depression was considered important by the patients. Although the results are reassuring, the group is heterogenic and extra attention could be given to the vulnerable young adults, recently transferred to the adult clinic. Screening is not the answer to every question regarding mental health but it is a reliable and easy way to bring these questions upon a regular basis. Our results stress the importance of a good and individualized transition from pediatric to adult care in order to provide a “smooth landing.” Both screening results and patient’s acceptance need to be followed up over time.

## Abbreviations

CES-D: Center for Epidemiologic Depression Scale
CF: Cystic fibrosis
CFTR: Cystic fibrosis transmembrane conductance regulator
GAD-7: Generalized Anxiety Disorder 7-item scale
GHQ-28: General Health Questionnaire
HADS: Hospital Anxiety and Depression Scale
ICMH: International Committee on Mental Health in Cystic Fibrosis
PHQ-9: Patient Health Questionnaire 9-item scale
